# Genicular Artery Embolization (GAE) for the Treatment of Advanced Knee Osteoarthritis in Selected Nonoperative Patients: A Pilot Study at 6 Months Follow-Up

**DOI:** 10.3390/jcm15156014

**Published:** 2026-08-02

**Authors:** Andrea Fidanza, Aurelio Picchi, Simone Ciaglia, Carmine Timpani, Luigi Zugaro, Gianfilippo Caggiari, Giandomenico Logroscino

**Affiliations:** 1 Unit of Orthopaedics, Department of Life Health & Environmental Sciences, University of L’Aquila, 67100 L’Aquila, AQ, Italy; giandomenico.logroscino@univaq.it; 2 Unit of Diagnostic and Interventional Radiology, SS Filippo e Nicola Hospital, 67051 Avezzano, AQ, Italy; simone.ciaglia@gmail.com (S.C.); timpanicarmine@gmail.com (C.T.); luigi.zugaro@virgilio.it (L.Z.); 3 Orthopaedic Department, Sassari University Hospital, 07100 Sassari, SS, Italy

**Keywords:** genicular artery, embolizzation, knee, osteoarthritis, no surgical treatment

## Abstract

**Background**: Genicular Artery Embolization (GAE) is an emerging minimally invasive procedure for the treatment of pain due to knee osteoarthritis (OA) in patients who are not candidates for joint replacement surgery. The aim of this study is to evaluate the clinical and functional outcomes of GAE performed with a temporary embolic agent and the persistence of its benefits up to 6 months of follow-up. **Methods**: In this prospective study, 15 consecutive patients (mean age 64.5 ± 6.7 years) with Kellgren–Lawrence grade III–IV knee OA, severe pain refractory to conservative treatments, and not eligible for joint replacement surgery were enrolled. All patients underwent GAE with selective embolization of hypervascular genicular branches using an imipenem/cilastatin suspension as a temporary embolic agent. Patients were evaluated before the procedure, on the first postoperative day, and at 3 and 6 months of follow-up using the Western Ontario and McMaster Universities Osteoarthritis Index (WOMAC), the Knee Injury and Osteoarthritis Outcome Score (KOOS), the Oxford Knee Score (OKS), and the Visual Analog Scale (VAS). **Results**: All clinical scores showed a significant improvement over time (*p* < 0.05). WOMAC decreased from 55.6 ± 7.3 to 16.3 ± 4.1 at 6 months; KOOS increased from 40.7 ± 8.1 to 70.3 ± 6.4; OKS improved from 18.4 ± 4.2 to 38.9 ± 4.6; VAS decreased from 7.8 ± 1.0 to 1.5 ± 0.8. No major complications were observed. Two patients (13.3%) developed a subcutaneous hematoma at the femoral access site, which resolved spontaneously. In two patients, pain recurred at the 1-month follow-up. **Conclusions**: GAE with a temporary embolic agent appeared to be a safe and effective procedure in improving pain and function in patients with advanced knee OA who are not candidates for joint replacement surgery. The clinical benefit was progressive and sustained up to 6 months, supporting the role of synovitis modulation as a therapeutic target.

## 1. Introduction

Knee osteoarthritis (OA) represents one of the main causes of chronic pain and functional disability in the adult and elderly population, with a significant impact on both patients’ quality of life and healthcare systems [1]. It is a degenerative joint disease characterized by progressive loss of hyaline cartilage, alterations of the subchondral bone, and a chronic inflammatory process of the synovial membrane, all of which contribute to pain, joint stiffness, and functional limitation [2,3].

The therapeutic approach to knee OA is initially conservative and includes non-pharmacological interventions such as weight reduction, modification of daily activities, and personalized exercise programs [4,5]. Pharmacological therapy mainly relies on nonsteroidal anti-inflammatory drugs (NSAIDs), administered either orally or topically, and intra-articular injections of corticosteroids or hyaluronic acid. However, these treatments often provide only temporary relief and do not modify disease progression [6].

In patients with persistent symptoms refractory to conservative therapies, prosthetic surgery—particularly total knee arthroplasty—currently represents the definitive treatment and the gold standard. This procedure has demonstrated high success rates in terms of pain reduction and functional recovery [7]. Nevertheless, a non-negligible proportion of patients are not eligible for surgery due to significant comorbidities, high perioperative risk, or other clinical contraindications. Additionally, some patients refuse surgery or require alternative therapeutic strategies while awaiting possible intervention.

In this context, genicular artery embolization (GAE) has emerged as a minimally invasive procedure aimed at treating pain associated with knee osteoarthritis [8]. The technique is based on the selective occlusion of arterial branches responsible for pathological neovascularization, leading to a reduction in synovial inflammation and pain. GAE therefore represents a potentially useful therapeutic option in patients who have an indication for surgery but are not candidates for prosthetic intervention, acting as a possible bridge between failure of conservative therapies and definitive treatment.

Several studies have shown that, in OA pathophysiology, the proliferation of newly formed microvessels accompanies and sustains synovial inflammation, contributing to the growth of nociceptive nerve fibers and thus to pain generation [9,10,11,12]. The aim of GAE is therefore to interrupt this pathological circuit by selectively occluding the small genicular branches supplying hyperemic areas identified during selective angiography [13].

The techniques used to achieve vascular occlusion can be divided into temporary and permanent embolization, depending on the material used and the duration of the embolic effect [14].

Temporary embolization is based on resorbable agents, including imipenem/cilastatin (IPM/CS) or calibrated gelatin particles, which may enhance the effect of IPM/CS in cases of marked hypervascularization [15]. Although IPM/CS is an antibiotic, when suspended with contrast medium it forms microcrystals capable of acting as embolic particles [16]. These crystals produce fine and selective occlusion of neovessels but gradually dissolve, allowing restoration of normal flow in non-pathological vessels over time. Similarly, gelatin particles exert a transient occlusive effect, as they are degraded by tissue enzymes [17]. This reversibility makes temporary embolic agents particularly useful in patients where inflammation control is desired without permanent vascular changes and with a theoretically lower risk of periarticular tissue ischemia.

Permanent embolization, on the other hand, uses microspheres composed of non-resorbable polymeric materials. Embozene microspheres consist of a hydrogel core (acrylic polymer) coated with Polyzene-F, whereas Embosphere microspheres are made of trisacryl-gelatin [18,19]. These are biocompatible, stable, and uniformly sized spherical particles designed for durable occlusion of small arterial branches responsible for synovial hypervascularization [20]. Their persistence allows prolonged interruption of inflammatory and angiogenic stimuli, with potential long-term clinical benefit. Due to their precise calibration, microspheres also provide high control over the level of occlusion and reduce the likelihood of unintended distal migration [21].

Regardless of the material used, embolization targets are not the main genicular arteries but rather their terminal branches responsible for inflammatory neoangiogenesis. The goal is not to compromise physiological vascularization of the knee, but to selectively reduce the pathological vascular network sustaining synovitis and pain progression [19]. This distinction is essential to ensure treatment efficacy and minimize ischemic complications.

Since it is a novel and ever-evolving process, there is not much research in the literature, and findings are contradictory. Data on functional outcomes, PROMs, and failures requiring surgery are also lacking.

The aim of the present study is to evaluate clinical and functional outcomes in a cohort of patients undergoing GAE using a temporary embolic agent. The study also aims to assess the persistence of the analgesic effect up to six months of follow-up.

## 2. Materials and Methods

This prospective pilot study included a series of consecutively enrolled patients who underwent embolization through selective catheterization of genicular arteries supplying areas of synovial hypervascularization, with injection of an IPM/CS suspension used as a temporary embolic agent. Procedures were performed at a specialized embolization center between July 2025 and December 2025. Patient recruitment is currently ongoing to reach the sample size established by the a priori power analysis. The results presented in this manuscript refer exclusively to patients who have completed the scheduled 6-month follow-up. Inclusion criteria were as follows: age > 40 years, persistent joint pain (VAS > 6) refractory to conservative treatments for at least 4–6 months, KL grade III–IV knee osteoarthritis, high surgical risk, and non-operable status. Exclusion criteria included ASA I–II, refusal of surgery despite eligibility, septic arthritis, avascular necrosis, autoimmune inflammatory diseases, and severe renal insufficiency.

Demographic data were collected, including age, sex, and side of the procedure. Body mass index (BMI) was calculated for each patient, and the degree of knee osteoarthritis was classified according to the Kellgren–Lawrence (KL) system. To identify the areas of most pain and to tailor embolization, each patient underwent a specialized orthopedic evaluation prior to the intervention, which included a history and physical examination. Every patient underwent MRI and X-ray; functional scores were assessed as well.

All patients were completely informed, in a clear and comprehensive way, of the procedure and possible conservative alternatives. Patients were treated according to the ethical standards of the Helsinki Declaration, and were invited to read, understand, and sign the informed consent form. The study protocol was approved by the Internal Review Board of the University of L’Aquila—Italy, ref. 9995 with identification number 09/2025.

### 2.1. Genicular Artery Embolization Procedure

All procedures were performed under fluoroscopic guidance by the same two experienced interventional radiologists (experience > 5 years) after appropriate clinical evaluation and orthopedic diagnostic assessment.

Vascular access was obtained via ultrasound-guided antegrade puncture of the contralateral common femoral artery, followed by placement of a 5 fr vascular sheath and intravenous heparin administration.

**Figure 1:**
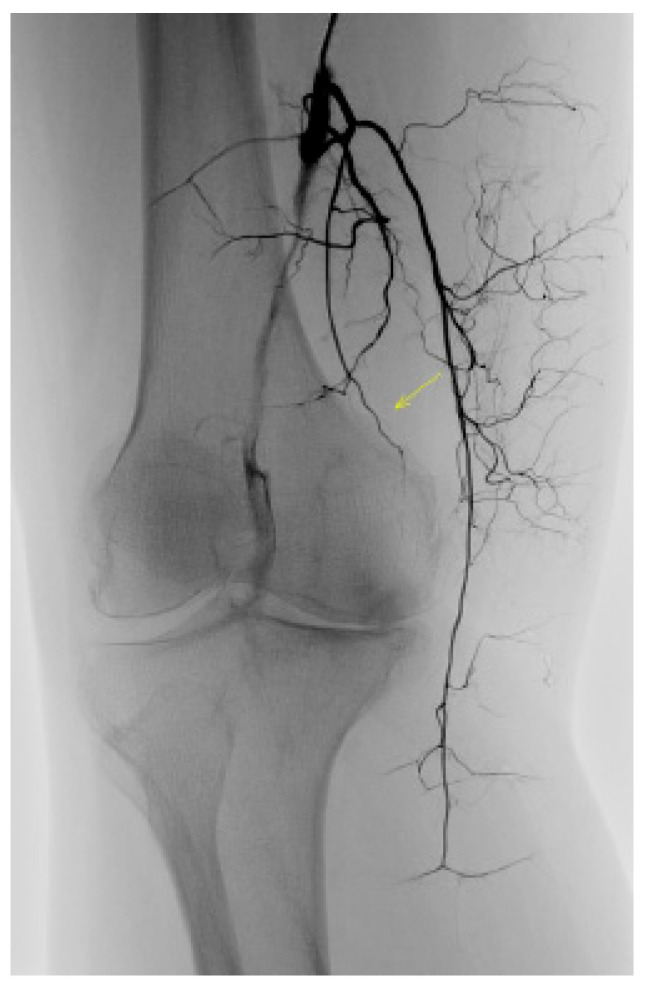
Frequency selective absorber (FSA) oblique angiography with enhanced descending genicular artery (arrow).

Digital subtraction angiography (DSA) of the femoropopliteal segment was then performed in anteroposterior and oblique projections (Figure 1).

**Figure 2:**
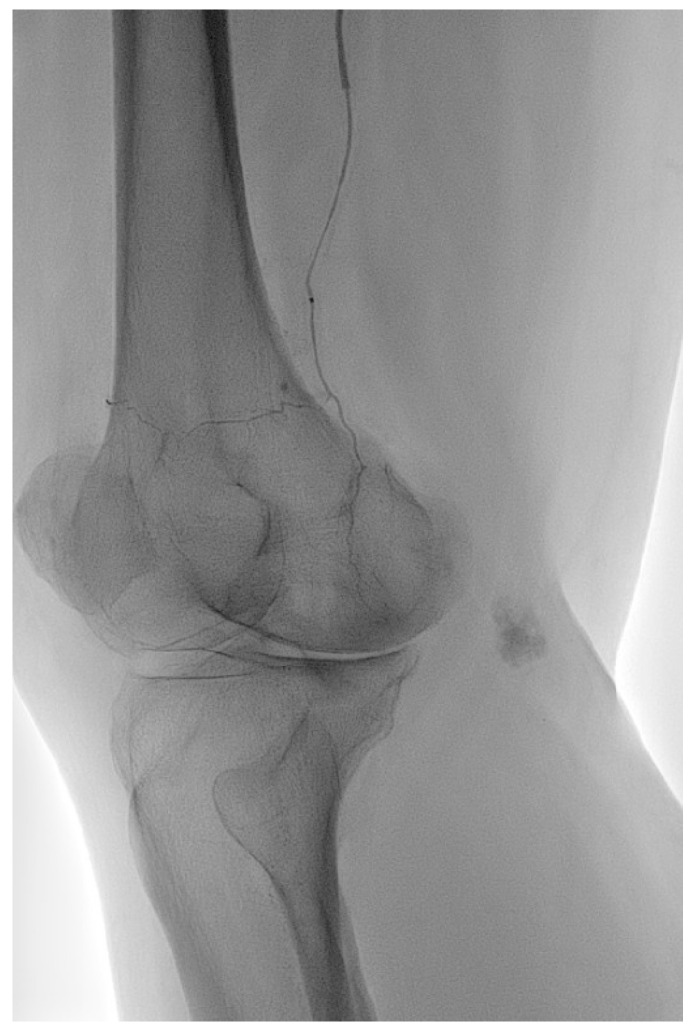
Descending genicular artery superselectively catheterized via coaxial with 1.9 fr catheter.

Genicular branches corresponding to clinically painful areas were superselectively catheterized using a 1.9 fr microcatheter (Figure 2).

Embolization was performed by slow infusion of an IPM/CS suspension in 10 cc of hydrophilic iodinated contrast medium agent. In patients with poorly localized pain, all genicular branches were selectively explored and embolized if pathological hypervascularization was observed.

**Figure 3:**
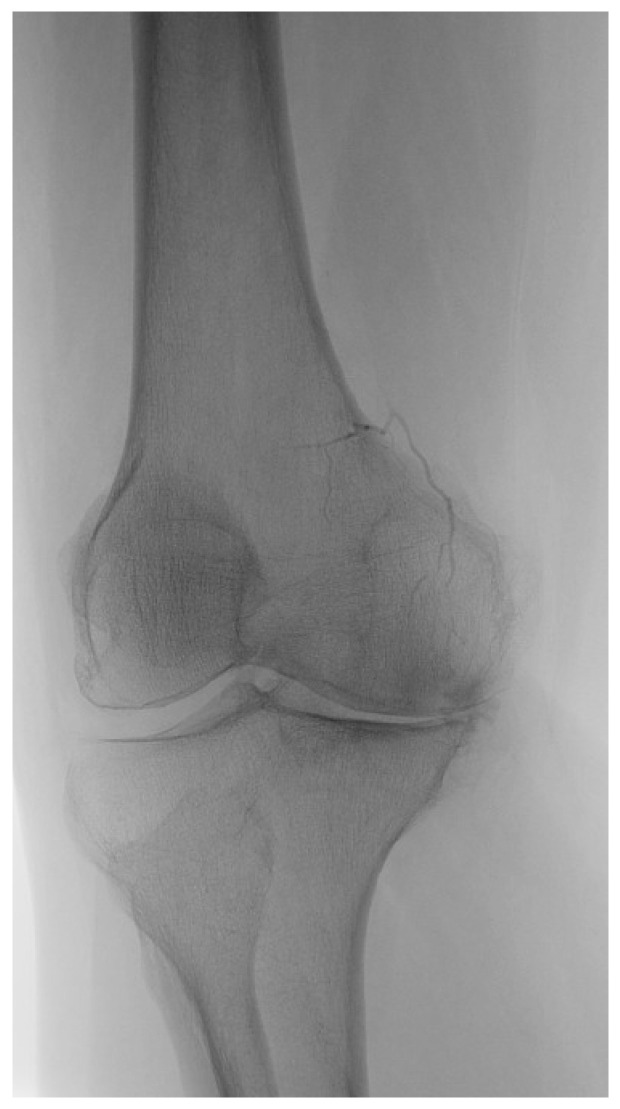
Flow stasis for 10 heartbeats observed under fluoroscopy, consequently leading to genicular artery embolization.

The technical embolization endpoint was flow stasis, considered as stasis for 10 heartbeats (Figure 3).

### 2.2. Post-Procedural Clinical Management

Patients were discharged the day after the procedure and scheduled for outpatient orthopedic follow-up. In cases of post-procedural pain related to embolization, analgesic therapy was prescribed for 1–2 weeks. Pre-procedural analgesic therapy was discouraged during follow-up unless pain persisted or worsened after GAE. Intra-articular injections were avoided throughout the observation period.

### 2.3. Patient-Reported Outcome Measures (PROMs)

Patients were evaluated before the procedure, the day after, at three months, and at six months using the Western Ontario and McMaster Universities Osteoarthritis Index (WOMAC), the Knee Injury and Osteoarthritis Outcome Score (KOOS), the Oxford Knee Score (OKS), and the Visual Analog Scale (VAS) for pain [22,23,24]. WOMAC assesses pain, stiffness, and physical function, with higher scores indicating worse symptoms. KOOS evaluates pain, symptoms, daily activities, sport function, and quality of life, with higher scores indicating better knee condition. OKS measures knee function and pain in daily life, where lower scores indicate worse outcomes. VAS is used to quantify pain intensity, with higher values corresponding to greater pain.

### 2.4. Statistical Analysis

Continuous variables were expressed as mean ± standard deviation (SD), while categorical variables were reported as absolute frequencies and percentages. Data distribution was assessed using the Shapiro–Wilk test. Given the limited sample size and the non-normal distribution of several outcome measures, non-parametric statistical methods were adopted. Longitudinal changes in clinical scores across the four assessment time points (baseline, 1 month, 3 months, and 6 months) were evaluated using the Friedman test for repeated measures. When a significant overall difference was observed, pairwise comparisons between baseline and follow-up assessments were performed using the Wilcoxon signed-rank test with Bonferroni correction for multiple comparisons. Exact *p*-values were reported whenever appropriate. A two-sided *p*-value < 0.05 was considered statistically significant. An a priori sample size calculation was performed using G*Power (version 3.1.9.7, Heinrich Heine University, Düsseldorf, Germany) based on the primary outcome (WOMAC score), assuming a two-sided α level of 0.05, a statistical power of 80%, and a large effect size according to previously published GAE studies. Patient recruitment is currently ongoing to achieve the target sample size determined by this analysis. The present manuscript reports an interim analysis including only patients who completed the predefined 6-month follow-up. Statistical analyses were performed using MedCalc^®^ Statistical Software, version 22.021 (MedCalc Software Ltd., Ostend, Belgium).

## 3. Results

A total of fifteen consecutive patients (six women and nine men) were treated and followed, with a mean age of 64.5 ± 6.7 years. Patients were classified according to KL as follows: two patients were classified as grade III and thirteen as grade IV. The mean BMI value was 30.5 ± 4.3.

A significant improvement was observed in all clinical evaluation scores, from preoperative assessment to three-month and six-month follow-up (Table 1). Two patients presented a subcutaneous hematoma at the femoral access site, which resolved spontaneously without the need for treatment (2/15, 13.33%).

### PROMs

The WOMAC score showed a progressive and statistically significant improvement over the course of follow-up, decreasing from a mean pre-procedural value of 55.6 ± 7.3 to 35.2 ± 8.1 the day after, 25.5 ± 5.8 at three months (*p* < 0.05), and 16.3 ± 4.1 at six months (*p* < 0.05).

The KOOS showed a progressive improvement over time, increasing from a mean pre-procedural value of 40.7 ± 8.1 to 51.5 ± 5.1 the day after the procedure, 58.5 ± 6.9 at three months (*p* < 0.05), and 70.3 ± 6.4 at six months (*p* < 0.05).

The OKS showed a progressive improvement over time, increasing from 18.4 ± 4.2 in the pre-procedural period to 25.4 ± 7.2 the day after, 28.6 ± 5.1 at three months (*p* < 0.05), and 38.9 ± 4.6 at six months (*p* < 0.05), indicating a significant functional recovery after the procedure.

**Figure 4:**
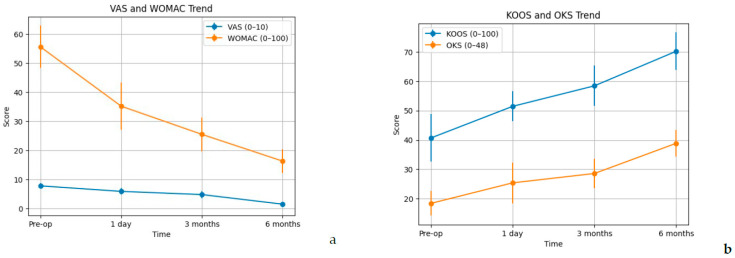
Western Ontario and McMaster Universities Osteoarthritis Index (WOMAC) and Visual Analogue Score (VAS) result in a downward trend immediately from the first day post-procedure (**a**). Similarly, Knee Injury and Osteoarthritis Outcome Score (KOOS) and Oxford Knee Score (OKS) also show a better score over time (**b**).

The VAS for pain also showed a significant improvement, with a reduction in the mean score from 7.8 ± 1.0 in the pre-procedural phase to 5.9 ± 1.1 the day after, 4.8 ± 1.2 (*p* < 0.05) at three months, and 1.5 ± 0.8 (*p* < 0.05) at six months (Figure 4).

No cases of skin necrosis, tissue ischemia, infections, neurological damage, or other ischemic complications were observed during the procedure or in the follow-up period. Two patients did not benefit from analgesic treatment, resulting in ongoing discomfort and discontent.

## 4. Discussion

The main finding of this study is that GAE significantly reduces pain in early follow-up, proving to be a promising and valid procedure. Consistent with Wang et al., this clinical impact is also confirmed by MR imaging, with a significant reduction in bone marrow edema after the procedure [25]. A first point of discussion concerns the type of embolic agent used in GAE. The studies by Okuno et al. and subsequently by Lee and Choi used IPM/CS as a temporary embolic agent, demonstrating a significant clinical improvement in pain and function with no major complications and a favorable safety profile [15,16,17]. In these studies, the reported complications were exclusively minor and transient, mainly hematomas at the access site or skin discoloration, all of which resolved spontaneously.

Conversely, more recent studies that employed permanent microspheres, such as those by Padia et al., Little et al., and Bagla et al., demonstrated good clinical efficacy, but at the cost of a higher risk of ischemic complications [18,19,20]. In particular, Padia and colleagues reported focal skin ulcerations in 18% of patients, asymptomatic bone infarctions in 5%, and one case of fat tissue necrosis, complications not observed with resorbable agents [20]. Similarly, the GENESIS study by Little et al. showed transient skin discoloration and a groin hematoma, although no documented osteonecrosis was observed [19]. Bhatia et al. directly compared IPM/CS and trisacryl-gelatin microspheres, reporting no statistically significant differences between the two materials [21]. However, the use of permanent agents was associated with a higher incidence of minor adverse events, such as transient skin changes and paresthesia, although no major complications were reported [26,27,28]. These data suggest that permanent embolization may guarantee a more prolonged effect, but temporary embolization offers a superior safety profile, particularly relevant in fragile, non-operable, or high-risk patients, such as those included in the present study.

A second element of discussion is represented by the duration of the clinical effect. Most studies on GAE demonstrate a significant improvement already at 1–3 months, with a peak of benefit between 6 and 12 months. However, long-term results remain heterogeneous and the duration of the clinical effect remains a matter of discussion [29].

According to Padia et al., 96% of respondents at three months maintained the benefit at twelve months, indicating a reasonable durability of the effect with persistent agents [20]. Similarly, studies with IPM/CS have shown persistence of benefit up to one year, although some patients required subsequent prosthetic intervention [18,19,20,30]. Bhatia et al. demonstrated persistence of the analgesic effect of GAE, both with temporary agents and permanent microspheres, suggesting that modulation of synovial neovascularization may induce a lasting biological effect, independent of the permanence of the embolic material [21].

In the present study, the analgesic and functional effect was maintained and further consolidated between 3 and 6 months, as demonstrated by the progressive trend of WOMAC, KOOS, OKS, and VAS. This finding supports the hypothesis that modulation of synovitis through selective embolization may induce a durable inflammatory reset, even in the presence of a temporary embolic agent, although the follow-up is short.

A further element of discussion concerns the dissociation between the severity of structural damage and pain intensity in patients with knee osteoarthritis [31]. In the present study, most patients had advanced OA (KL IV), yet still showed significant clinical improvement after GAE, suggesting that osteoarthritic pain does not depend exclusively on cartilage loss: in fact, post-procedure radiographic investigations were not foreseen as they would have been superfluous [32,33]. This finding supports the central role of synovitis and pathological neoangiogenesis as the main drivers of pain [34]. Longitudinal studies with advanced imaging have shown that variation in synovitis, more than cartilage loss, correlates with pain variation in patients with knee OA, suggesting that inflammatory joint processes represent a key therapeutic target [35,36]. GAE, by acting selectively on the vascular-inflammatory component, allows symptomatic improvement even in the absence of structural modifications, confirming the validity of a biological rather than purely mechanical approach to the treatment of osteoarthritis [19]. This concept reinforces the role of GAE as a targeted and complementary analgesic treatment, rather than a structural alternative to prosthetic surgery [18,19,20,21].

A crucial aspect, often underestimated in previous studies, is the role of the multidisciplinary approach in the management of patients with knee OA candidates for GAE. In our experience, to get the correct indication and therefore achieve the best results, patient selection was performed by the orthopedic surgeon, who is familiar with the natural history of the disease, available therapeutic options, and the correct timing of prosthetic surgery [18]. In our organizational model, diagnosis, staging according to KL, and the indication for possible treatment are formulated by the orthopedic surgeon, while the indication and eligibility for the embolization procedure are determined by the radiologist, with specific expertise in the selection of pathological vascular branches and in the prevention of non-target embolization. Clinical follow-up is again entrusted to the orthopedic surgeon, supported by the radiologist for any technical concerns, with evaluation of symptom evolution, functional response, and possible osteoarthritic progression. This collaborative model allows integration of GAE into the patient’s therapeutic pathway, placing it as an intermediate option between failure of conservative therapies and prosthetic surgery, especially in patients not eligible for intervention.

The present study has some limitations. A major limitation of this study is the lack of a control or sham-treated group, which precludes definitive conclusions regarding treatment efficacy, as the observed improvements may have been influenced by placebo effects, natural disease variability, or regression toward the mean. Further controlled studies are warranted to confirm these preliminary findings. The small sample size reduces the generalizability of the results and does not allow direct comparisons with other therapeutic strategies. The follow-up limited to 6 months does not allow definitive conclusions regarding the long-term duration of the clinical effect, and it is also not possible to verify the actual duration of embolization. Finally, the absence of a structured post-procedural radiological evaluation does not allow correlation between clinical improvement and possible anatomical changes in the synovium or subchondral bone.

The strength of the study lies in the fact that it is the first report with clinical impact using PROMs with rating scales recognized by the international orthopedic community. Furthermore, the pilot approach of the interdisciplinary assessment model proposed for the correct procedure recommendation is unique in the literature, as the technique seems to be carried out and acknowledged exclusively by the radiology community: integrating knowledge from many fields is the only way to attain the greatest outcomes for personalized treatment medicine.

## 5. Conclusions

The results of the present study suggest that GAE with a temporary embolic agent could represent a safe and effective procedure for the treatment of pain due to knee osteoarthritis in patients not eligible for prosthetic surgery. Even if the observed clinical improvement was progressive and sustained up to 6 months, the results should be interpreted as a preliminary observational series rather than as proof of absolute efficacy.

The favorable safety profile, with absence of major complications, supports the use of temporary embolization in fragile patients or those at high surgical risk. Randomized prospective studies with larger sample sizes and longer follow-up are necessary to define the definitive role of GAE, the optimal type of embolization, and its duration in the treatment of knee osteoarthritis.

The integration of GAE into a multidisciplinary pathway allows optimization of patient selection and clinical follow-up.

## Figures and Tables

**Table 1:** Results of clinical evaluation scores.

	Clinical Evaluation		*p*-Value
	Pre-Op	Post-Op	
		1 d	
		3 m	
		6 m	
WOMAC	55.6 ± 7.3	35.2 ± 8.1	<0.05
		25.5 ± 5.8	<0.05
		16.3 ± 4.1	<0.05
KOOS	40.7 ± 8.1	51.5 ± 5.1	<0.05
		58.5 ± 6.9	<0.05
		70.3 ± 6.4	<0.05
OKS	18.4 ± 4.2	25.4 ± 7.0	<0.05
		28.6 ± 5.1	<0.05
		38.9 ± 4.6	<0.05
VAS	7.8 ± 1.0	5.9 ± 1.1	<0.05
		4.8 ± 1.2	<0.05
		1.5 ± 0.8	<0.05

## Data Availability

The original contributions presented in this study are included in the article. Further inquiries can be directed to the corresponding authors.
